# In Macrophages, Caspase-1 Activation by SopE and the Type III Secretion System-1 of *S*. Typhimurium Can Proceed in the Absence of Flagellin

**DOI:** 10.1371/journal.pone.0012477

**Published:** 2010-08-30

**Authors:** Claudia Hoffmann, Marlies Galle, Sabrina Dilling, Rina Käppeli, Andreas J. Müller, Pascal Songhet, Rudi Beyaert, Wolf-Dietrich Hardt

**Affiliations:** 1 Institute of Microbiology, D-BIOL, ETH Zürich, Zürich, Switzerland; 2 Unit for Molecular Signal Transduction in Inflammation, Department for Molecular Biomedical Research, VIB, Ghent, Belgium; 3 Department of Molecular Biology, Ghent University, Ghent, Belgium; Universidad Nacional, Costa Rica

## Abstract

The innate immune system is of vital importance for protection against infectious pathogens. Inflammasome mediated caspase-1 activation and subsequent release of pro-inflammatory cytokines like IL-1β and IL-18 is an important arm of the innate immune system. *Salmonella enterica* subspecies 1 serovar Typhimurium (*S*. Typhimurium, SL1344) is an enteropathogenic bacterium causing diarrheal diseases. Different reports have shown that in macrophages, *S*. Typhimurium may activate caspase-1 by at least three different types of stimuli: flagellin, the type III secretion system 1 (T1) and the T1 effector protein SopE. However, the relative importance and interdependence of the different factors in caspase-1 activation is still a matter of debate. Here, we have analyzed their relative contributions to caspase-1 activation in LPS-pretreated RAW264.7 macrophages. Using flagellar mutants (*fliGHI*, *flgK*) and centrifugation to mediate pathogen-host cell contact, we show that flagellins account for a small part of the caspase-1 activation in RAW264.7 cells. In addition, functional flagella are of key importance for motility and host cell attachment which is a prerequisite for mediating caspase-1 activation via these three stimuli. Using site directed mutants lacking several T1 effector proteins and flagellin expression, we found that SopE elicits caspase-1 activation even when flagellins are absent. In contrast, disruption of essential genes of the T1 protein injection system (*invG*, *sipB*) completely abolished caspase-1 activation. However, a robust level of caspase-1 activation is retained by the T1 system (or unidentified T1 effectors) in the absence of flagellin and SopE. T1-mediated inflammasome activation is in line with recent work by others and suggests that the T1 system itself may represent the basic caspase-1 activating stimulus in RAW264.7 macrophages which is further enhanced independently by SopE and/or flagellin.

## Introduction

Caspase-1 is a central switch triggering inflammation and mounting innate immune defenses. Activation of caspase-1 occurs in a multiprotein complex termed the “inflammasome” [1]. The inflammasome is composed of NOD-like receptors (NLRs) such as Nalp3, NLRC4/IPAF, and Nalp1, and the adaptor ASC (apoptosis-associated speck-like protein containing a CARD), that assemble in response to intracellular presence of danger- or pathogen-associated molecular patterns (DAMPs or PAMPs, respectively) (for review see [2]). Pro-caspase-1 is recruited to either of the different inflammasome complexes and, following autoproteolytic cleavage, becomes activated. Active caspase-1 processes pro-interleukin-1β and pro-interleukin-18 into their mature forms IL-1β and IL-18 which are subsequently secreted from the cell and induce a strong pro-inflammatory response, thereby retarding systemic spread of numerous pathogens [3], [4]. Recently, it has been found that active caspase-1 is itself secreted from cells together with a number of different factors, e.g. IL-1α whose secretion is also regulated by caspase-1 [5].

The inflammasome can be activated by such diverse stimuli as pore-forming toxins, extracellular ATP, cytosolic presence of DNA, uric acid crystals, or bacterial flagellin [6]. Often, a particular stimulus activates caspase-1 via just one particular inflammasome. In the case of pathogenic bacteria, one particular pathogen may release more than one stimulus and may activate one or more different inflammasomes. In macrophages, *Salmonella enterica* subspecies I serovar Typhimurium (*S*. Typhimurium) is sensed mainly via the NLRC4/IPAF inflammasome (for review see [7]). In recent publications, three different proteins released by *S*. Typhimurium have been shown to activate caspase-1: a. Several reports have shown that bacterial flagellin may act as a potent inducer of the NLRC4/IPAF inflammasome [8], [9], [10]. It is thought that caspase-1 activation requires injection of flagellin into the host cell cytosol via the type three secretion system 1 (T3SS-1; termed T1 in this paper) [10]. b. Miao et *al*. (2010) identified a component of the basal body inner rod of the T3SS of various pathogens, including the T1 apparatus protein PrgJ from *S*. Typhimurium, as a stimulator of the NLRC4/IPAF inflammasome [11]. A *S*. Typhimurium mutant lacking flagellin activated caspase-1 in wild type macrophages, whereas a *prgJ* deletion mutant did not, underlining the importance of a functional *Salmonella* T1 system for caspase-1 activation [11]. c. Recently, we found that the *S*. Typhimurium effector protein SopE, which is injected into the host cell cytoplasm via the T1 system activates caspase-1 in different cell types, including macrophages [12]. SopE-mediated caspase-1 activation was attributable to the guanine nucleotide exchange factor (GEF) activity of SopE [13]. In this way, SopE activates host cellular RhoGTPases and thereby triggers host cell invasion and caspase-1 activation in parallel [12]. The presence of at least three different stimuli raised the question whether each stimulus by itself was sufficient or whether they must cooperate for activating caspase-1 in macrophages. This question has not been adequately addressed so far, i.e. in the case of SopE.

Here, we analyzed whether SopE can activate macrophage caspase-1 in the absence of flagellin. This was not addressed rigorously in the past because of the dual function of flagellin in the infection process. In addition to serving as an inflammasome activating stimulus, flagellin is required for propelling the pathogen towards the host cell. Thus, flagellin-deficient mutants of *S*. Typhimurium cannot activate caspase-1 directly and they fail to deploy T1 or inject SopE, because they do not efficiently reach the host cell. To analyze contributions of the T1 system and of SopE to flagellin-independent activation of caspase-1, we used amotile *S*. Typhimurium mutants that either lack expression of both *S.* Typhimurium flagellins (Δ*fliGHI* or Δ*fliC*Δ*fljB*), or still express flagellin monomers but do not assemble functional flagella (Δ*flgK*). The motility defect was compensated to a large extent by centrifugation which established efficient host cell contact even of amotile mutants. Our data show that even in the absence of flagellin, T1-dependent stimuli (most likely the translocon itself) and SopE mediate caspase-1 activation.

## Methods

### Bacterial strains and plasmids

All *S*. Typhimurium strains used were isogenic derivatives of SL1344 of *Salmonella enterica* subspecies I serovar Typhimurium (*S.* Typhimurium) (Table 1) [14]. Strains SB161 (Δ*invG*) [15], M566 (Δ*sopB* Δ*sipA* Δs*opE* Δ*sopE2*) [16], SB169 [17], M1335 [12], M1336 [12], and M913 (*fliGHI::Tn10*) [18] have been described previously. M562 carrying in-frame deletions of *sopB* and *sipA* was obtained by allelic exchange in the chromosome of M509 (Δ*sopB*) [19] by using the suicide vector pM585 as described in [16].

**Table 1:** Strains used in this study.

Designation	Strain	Characteristics	Genotype	Reference
**WT**	SB300	expresses FliC and FljB	wildtype *S*. Typhimurium SL1344	[14]
**^a^ WT^M−F−^**	M913	motility^−^, flagellin^−^	*fliGHI*::Tn10	[18]
**^b^ WT^M−F+^**	M2422	motility^−^, flagellin^+^	*flgK*::Tet^R^	this study
**SopE/E2**	M562		Δ*sipA* Δ*sopB*	this study
**SopE/E2^M−F−^**	M2414	motility^−^, flagellin^−^	Δ*sipA* Δ*sopB fliGHI*::Tn10	this study
**SopE/E2^M−F+^**	M2425	motility^−^, flagellin^+^	Δ*sipA* Δ*sopB flgK*::Tet^R^	this study
**SopE**	M2421		Δ*sipA* Δ*sopB* Δ*sopE2*	this study
**SopE^ M−F−^**	M2432	motility^−^, flagellin^−^	Δ*sipA* Δ*sopB* Δ*sopE2 fliGHI*::Tn10	this study
**SopE^ M−F+^**	M2436	motility^−^, flagellin^+^	Δ*sipA* Δ*sopB* Δ*sopE2 flgK*::Tet^R^	this study
**T1-**	SB161	no T3SS-1	Δ*invG*	[15]
**T1-^M−F−^**	M2405	no T3SS-1, motility^−^, flagellin^−^	Δ*invG fliGHI*::Tn10	this study
**T1-^M−F+^**	M2423	no T3SS-1, motility^−^, flagellin^+^	Δ*invG flgK*::Tet^R^	this study
**WT_TEM_**	M2407		*sopE*::*sopE* ^M45^ *-tem-1*	this study
**WT_TEM_^M−F−^**	M2410	motility^−^, flagellin^−^	*sopE*::*sopE* ^M45^ *-tem-1 fliGHI*::Tn10	this study
**WT_ TEM_^M−F+^**	M2417	motility^−^, flagellin^+^	*sopE*::*sopE* ^M45^ *-tem-1 flgK*::Tet^R^	this study
**SopE/E2_TEM_**	M2409		Δ*sipA* Δ*sopB sopE*::*sopE* ^M45^ *-tem-1*	this study
**SopE/E2_TEM_^M−F−^**	M2416	motility^−^, flagellin^−^	Δ*sipA* Δ*sopB sopE*::*sopE* ^M45^ *-tem-1 fliGHI*::Tn10	this study
**SopE/E2_TEM_^M−F+^**	M2419	motility^−^, flagellin^+^	Δ*sipA* Δ*sopB sopE*::*sopE* ^M45^ *-tem-1 flgK*::Tet^R^	this study
**T1-_TEM_**	M2408	no T3SS-1	Δ*invG sopE*::*sopE* ^M45^ *-tem-1*	this study
**T1-_TEM_^M−F−^**	M2411	no T3SS-1, motility^−^, flagellin^−^	Δ*invG sopE*::*sopE* ^M45^ *-tem-1 fliGHI*::Tn10	this study
**T1-_TEM_^M−F+^**	M2418	no T3SS-1, motility^−^, flagellin^+^	Δ*invG sopE*::*sopE* ^M45^ *-tem-1 flgK*::Tet^R^	this study
**Δ4**	M566	lacks four T1 effector proteins	Δ*sopE* Δ*sopE2* Δ*sipA* Δ*sopB*	[16]
**Δ4^M−F−^**	M2406	lacks four T1 effector proteins, motility^−^, flagellin^−^	Δ*sopE* Δ*sopE2* Δ*sipA* Δ*sopB fliGHI*::Tn10	this study
**Δ4^M−F+^**	M2424	lacks four T1 effector proteins, motility^−^, flagellin^+^	Δ*sopE* Δ*sopE2* Δ*sipA* Δ*sopB flgK*::Tet^R^	this study
**Δ8**	M2400	lacks six T1 effector proteins, SpvB and SpvC	Δ*sopE* Δ*sopE2* Δ*sipA* Δ*sopB* Δ*sopA* Δ*sptP* Δ*spvB* Δ*spvC*	this study
**Δ8^M−F−^**	M2433	lacks six T1 effector proteins, SpvB and SpvC, motility^−^, flagellin^−^	Δ*sopE* Δ*sopE2* Δ*sipA* Δ*sopB* Δ*sopA* Δ*sptP* Δ*spvB* Δ*spvC fliGHI*::Tn10	this study
**Δ8^M−F+^**	M2437	lacks six T1 effector proteins, SpvB and SpvC, motility^−^, flagellin^+^	Δ*sopE* Δ*sopE2* Δ*sipA* Δ*sopB* Δ*sopA* Δ*sptP* Δ*spvB* Δ*spvC flgK*::Tet^R^	this study
**ΔSipB**	SB169	does not form a translocon pore	*sipB*::*aphT*	[48]
**SopE^M45^**	M1335	SopE^M45^	*sopE*::*sopE* ^M45^ Δ*sopE2* Δ*sipA* Δ*sopB sseD::aphT*	[12]
**SopE^M45^G168V**	M1336	catalytically inactive SopE^M45^G168V	*sopE*::*sopE(G168V)* ^M45^ Δ*sopE2* Δ*sipA* Δ*sopB sseD::aphT*	[12]

a. M**^−^**F**^−^**: no expression of flagellin, no flagella (amotile).

b. M**^−^**F**^+^**: expression of flagellins (FliC and FljB), no assembly of flagella (amotile).

M2400 carrying in-frame deletions of *sipA*, *sopA*, *sopE*, *sopE2*, *sopB*, *sptP*, *spvB*, and *spvC* was generated by sequential allelic exchange in the chromosome of M712 [20] by using the suicide plasmids pM1315 (see below) and pM1664 (see below). The knockouts were verified by PCR.

To obtain M2421, the primers 5′-ATGACTAACATAACACTATCCACCCAGCACTACAGAATCGTGTAGGCTGGAGCTGCTTC-3′ and 5′-TCAGGAGGCATTCfTGAAGATACTTATTCGCAATATTTTCATGGGAATTAGCCATGGTCC-3′ were used which have homology to the 5′ and 3′ coding regions of *sopE2* and the chloramphenicol-resistance cassette of pKD3 [21]. Deletion of *sopE2* was introduced in M705 (Δ*sopB* Δ*sopE sseD::aphT*, derivative of M509) [16] using the lambda Red-recombinase method [21]. M705 was constructed by P22 HT105/1 *int-201* transduction [22]of *sseD::aphT* allele from MvP101 (*S.typhimurium* ATCC14028 derivative) [23] into M560 (Δ*sopB* Δ*sopE*; Ehrbar and Hardt, unpublished). M560 was constructed by double crossover recombination with pM509 (Ehrbar and Hardt, unpublished) using M509 (Δ*sopB*) [19]. The *sopE2* deletion was transduced by P22 HT105/1 *int-201*-mediated transduction into M562, and the plasmid pCP20 encoding for Flp recombinase was introduced to remove the Cm-resistance cassette, yielding M2421. Excision of the gene was verified by PCR.

M913 has been described previously and was generated by P22 HT105/1 *int-201*-mediated transduction of *fliGHI::Tn10* from SB245 (described in [18]). M2405, M2406, M2414, M2432, and M2433 were constructed in analogy to M913 by P22 HT105/1 *int-201*-mediated transduction of *fliGHI::Tn10* from SB245 into recipient strains SB161, M566, M562, M2421, and M2400, respectively. Lack of flagellin expression was verified by Western Blot analysis and lack of motility on motility agar plates.

*flgK* was deleted in SL1344 (SB300) according to the method of Datsenko & Wanner [21] by insertion of a Cm^R^-cassette that was amplified using the forward primer 5′- CGCTGCCGATAACAACGAGTATTGAAGGATTAAAAGGAACCATCATAATATGAATATCCTCCTTAGTT-3′ and the reverse primer 5′- GTTCGTACATCATCTGGGTACTGATACGCATGTCATCCTTCTCCTTGTGTAGGCTGGAGCTGCTTC-3′ into the *flgK* locus (*flgK::Cm^R^*). Subsequently, strains M2417, M2418, M2419, M2422, M2423, M2424, M2425, M2436, and M2437 were generated by P22 HT105/1 *int-201*-mediated transduction of the *flgK::Cm^R^* mutation from SL1344 *flgK* into recipient strains M2407, M2408, M2409, SB300, SB161, M566, M562, M2421, and M2400, respectively. Lack of motility of *flgK* mutants was tested on motility agar.

Strains encoding *sopE^M45^-tem-1* (amino acid sequence of M45 epitope tag: MDRSRDRLPPFETETRIL, [24]) (M2407, M2408, M2409, M2410, M2411, and M2416) were generated by integration of the suicide plasmid pM2401 (see below) into the chromosome of the recipient strains SB300, SB161, M562, M913, M2405, M2414, and M2421, respectively, by conjugational transfer.

All strains were verified by PCR and Western blot analysis.

For construction of the suicide plasmid pM2401, a *sopE^M45^*-fragment (lacking the N-terminus) was PCR-amplified from genomic DNA of SB875 [25] using the forward primer 5′-CAGACGTCAACAGGAAACCACACTAC-3′ and the reverse primer 5′-CTGGATCCGCGTCTCTGTC-3′. The resulting PCR product was then cloned into the *Aat*II- and *BamH*I-digested backbone of pM1132 [26] carrying the *tem-1* gene. The resulting *sopE^M45^*-*tem-1* genetic fusion was isolated from pM2400 by sequential digests with *EcoR*I and *Cla*I and subcloned into *EcoR*I- and *Cla*I-digested pM706 [27], yielding pM2401.

The suicide plasmid M1315 for deletion of *sptP* was constructed as follows: primers with homology to the upstream region (forward primer with *Xma*I site overhang: 5′-TTTCCCGGGAAAGATGCGATGAATA-3′, reverse primer with *Spe*I site overhang: 5′-TTTACTAGT CAATTTTCTCTCCTCATACTTTAGCA-3′) and the downstream region of *sptP* (forward primer with *Spe*I site overhang: 5′-TTTACTAGTAAGCCCAGTTGCTTATGACG-3′, reverse primer with *Not*I site overhang: 5′-TTTGCGGCCGCGCAGGGATCACTAAGCTGT-3′) were used for amplification by PCR and sequential cloning of the resulting fragments into pBluescript SKII+ (Stratagene). The insert was excised with *Xma*I and *Not*I and subcloned into pSB890 [15], yielding suicide plasmid pM1315.

For construction of the suicide plasmid pM1664, the 5′ end of *spvB* and the 3′ end of *spvC* were amplified by PCR from SL1344 plasmid 1 (forward primer for *spvB* with *Xma*I site overhang: 5′- CTTCCCGGGTCAGTCTTCAGGATTTCATTC-3′, reverse primer for *spvB* with *Spe*I site overhang: 5′-CCCACTAGTCAACATACACTATCTCCTGAAAC-3′; forward primer for *spvC* with *Spe*I site overhang: 5′-CCCACTAGTCAGAGTAAGTATGGGTTTGGG-3′, reverse primer for *spvC* with *Not*I site overhang: 5′-CTTGCGGCCGCAGGGTTTACAGCGGATCTTG-3′). The resulting fragments were sequentially cloned into pBluescript SKII+ (Stratagene), cut with *Xma*I and *Not*I and ligated into pSB890 [15], yielding suicide plasmid pM1664.

### Western Blot Analysis of flagellin and SopE

For Western Blot of bacterial cells and supernatants, aliquots were taken from T1-induced subcultures of *S*. Typhimurium strains. Bacteria were pelleted by centrifugation and supernatants were collected and further purified by a second centrifugation step. Pellets and TCA-precipitated supernatants were separated by 12% SDS-PAGE. FliC and FljB were detected with a polyclonal α-H1,2 antiserum (SIFIN, Germany). SopE was detected with a polyclonal α-SopE antiserum [28].

### Cell culture and infection experiments

RAW264.7 macrophage-like cells (ATCC No. TIB-71) were cultured in RPMI (10% FCS, 1% L-Glutamine) at 37°C/5% CO_2_. For infection experiments for analysis of LDH and Il-1 release, cells were seeded in 96 well plates the day before. 20′000 RAW264.7 cells were seeded per well and were pre-stimulated with 1 µg/ml *E. coli* LPS (List Biological Laboratories, California) overnight to induce expression of pro-IL-1β. This treatment did not affect responsiveness in the LDH release assay (data not shown). To induce T1 expression, *S*. Typhimurium strains were grown in LB (0.3 M NaCl) overnight at 37°C and subcultivated for 4 hours as described (Hapfelmeier et al., 2004). Bacteria were diluted in cell culture medium to the desired MOI and added to the cells. Where indicated, cell plates were centrifuged (500× g/10 min) to enhance contact of bacteria with the cells. Infected cells were incubated at 37°C for 30 min. Gentamycin (400 µg/ml) was added and cells were incubated for one additional hour at 37°C. Plates were then centrifuged (250× g/5 min) and supernatants were collected for measuring LDH release and mature IL-1, respectively.

### LDH release

Lactate dehydrogenase (LDH) was measured using the CytoTox 96® Non-Radioactive Cytotoxicity Assay (Promega) according to the manufacturer's instructions. The relative amount of released LDH was calculated as follows: % released LDH (sample)  =  (sample - medium background)/(total LDH - medium background) ×100.

### IL-1 bioassay

For detection of mature IL-1, supernatants collected from infected cells were supplemented with chloramphenicol (30 µg/ml) and incubated for 1 h at 37°C. Samples were stored at −80°C until they were analyzed by IL-1 bioassay as described before [29].

### Analysis of effector translocation by TEM-1 beta-lactamase assay

Translocation of SopE^M45^ into macrophages was analyzed using the TEM-1 β-lactamase reporter assay as described previously [30]. Briefly, 10′000 RAW264.7 cells were seeded in 96-well plates (µ-clear bottom, half area, Greiner Bio One) one day before and pre-stimulated overnight with 1 µg/ml *E. coli* LPS (List Biological Laboratories, California). If not stated otherwise, cells were infected for 60 min at a MOI of 150 with T1-induced cultures expressing SopE^M45^-TEM-1. Where indicated, cell plates were centrifuged at 500× g for 10 min directly after addition of bacteria. Following incubation, cells were washed with HBSS (400 µg/ml gentamycin) and incubated for 30 min at room temperature. Next, cells were loaded with 1 µg/ml CCF2-AM dye for 90 min at room temperature according to the manufacturer's instructions (Invitrogen). Cleavage of the internalized CCF2 dye by the translocated SopE^M45^-TEM-1 fusion protein was monitored by quantification of the fluorescence signal using a Victor3 microplate reader (PerkinElmer) with excitation at 405 nm and emissions at 460 nm (blue fluorescence) and 535 nm (green fluorescence). The emission ratio of 460/535 nm reflects translocation of the TEM-1 fusion protein.

### Bacterial attachment

For analyzing adherence of bacteria to cells, 10′000 RAW264.7 cells were seeded in 96-well plates (µ-clear bottom, half area, Greiner Bio One) one day before infection and pre-stimulated overnight with 1 µg/ml *E. coli* LPS (List Biological Laboratories, California). Cells were infected with T1-induced cultures (MOI = 150) and plates were either centrifuged at 500× g for 10 min directly after addition of bacteria or left at room temperature for 10 min. Plates were then incubated at 37°C for 6 min before they were washed three times with RPMI containing 0.5% BSA and 400 µg/ml Gentamycin. Subsequently, cells were fixed with 4% paraformaldehyde (in PBS with 4% Sucrose) for 15 min at room temperature. For visualization of extracellular bacteria, cells were incubated with a polyclonal α-*Salmonella*-LPS antibody (*Salmonella* O Antiserum Factors 4,5, Difco, Kansas) and a secondary α-rabbit-Cy5 antibody (Jackson, Pennsylvania). After permeabilization, nuclei were stained with DAPI (Sigma-Aldrich) and F-actin was stained with Phalloidin-TRITC (Sigma-Aldrich). Microscopy images were acquired with an ImageXpress Micro microscope (Molecular Devices) with a 10x objective. For quantification of cells associated with bacteria, cells from four single images from two independent experiments were evaluated (∼150–350 cells/image).

### Statistical analysis

Statistical analysis was performed using the Mann-Whitney U test (Prism Version 5) and the paired t-test (Prism Version 5). P-values less than 0.05 (two-tailed) were considered statistically significant.

## Results

In macrophages, *S*. Typhimurium SL1344 can trigger the inflammasome and subsequent caspase-1 activation via flagellin [8], [9], [10]. More recent reports show that, besides flagellin, components of the T1 apparatus as well as the T1 effector protein SopE may act as stimulators of caspase-1 [11], [12]. However, different macrophage cell models have been used to identify the effects of the different stimuli. Here, we wanted to determine the relative contributions of the three types of stimuli to caspase-1 activation in a well established macrophage cell line, i.e. RAW264.7 cells.

### Caspase-1 is activated in the absence of flagellin

**Figure 1:**
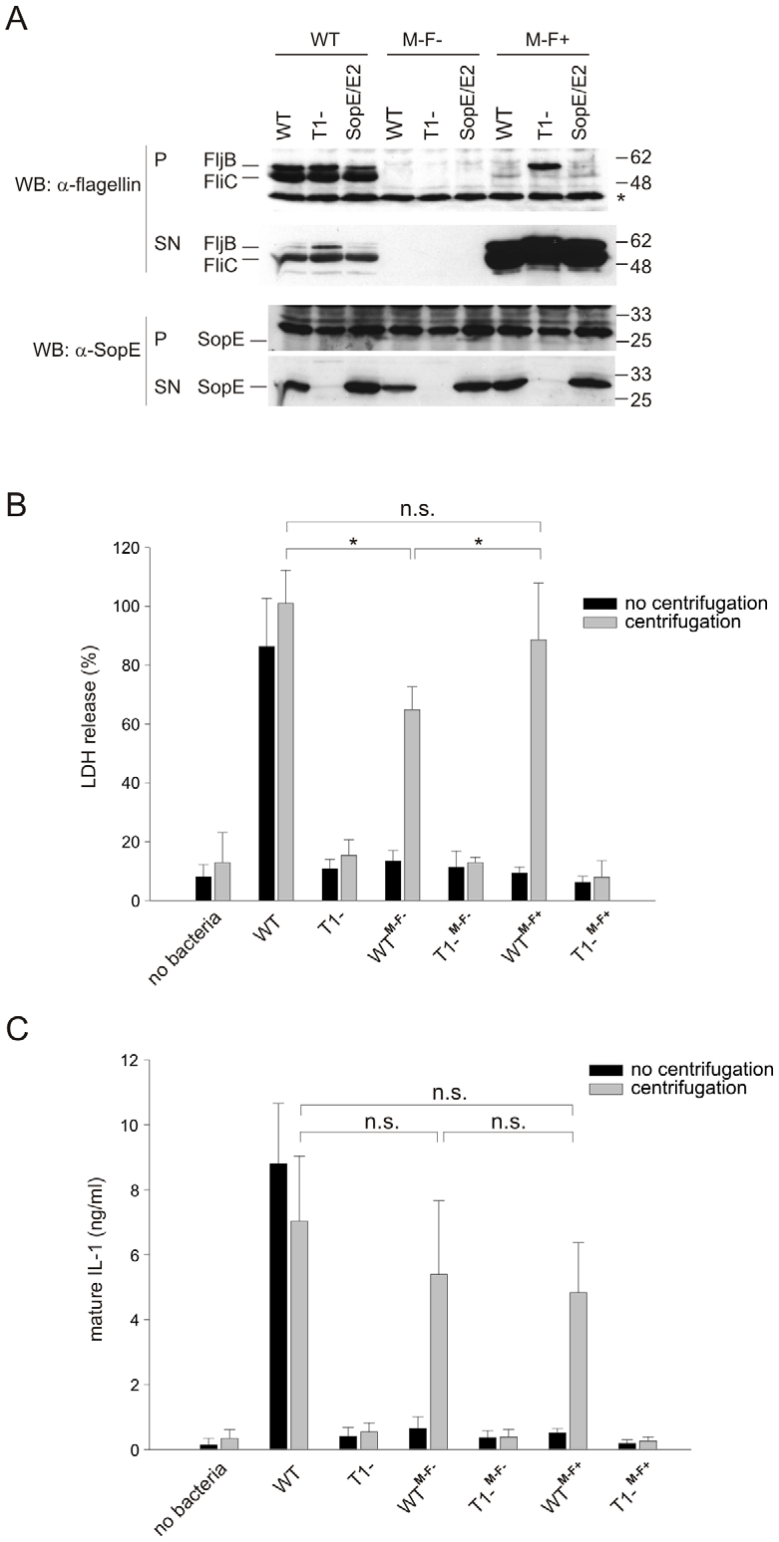
IL-1 maturation and LDH release induced by flagellin-deficient *S*. Typhimurium. **A**) Western Blot analysis of *Salmonella* flagellins (FliC and FljB) and the T1 effector SopE in lysates (P) and supernatants (SN) of flagella wildtype strains and Δ*fliGHI* (M−F−). WT: wildtype, T1^−^: no T3SS-1, SopE/E2: Δ*sipA* Δ*sopB*; *:unspecific band as loading control. **B**) Flagellin-deficient *S*. Typhimurium induce LDH release from LPS-pretreated RAW264.7 macrophages. Infection was performed with the indicated *S*. Typhimurium strains (MOI 150) either without (black bars) or with centrifugation (grey bars) of cell plates. **C**) Release of mature IL-1 after infection of LPS-pretreated RAW264.7 macrophages with flagellin-deficient *S*. Typhimurium (Δ*fliGHI,* M−F−) following centrifugation. Experiments were performed in triplicate; mean +/− SD. n.s.: not significant; *: p-value ≤0.05 (Mann-Whitney U test).

First, we verified that caspase-1 activation by *S*. Typhimurium can take place in the absence of flagellin. To this end, we used *fliGHI* mutants which lack expression of both *S*. Typhimurium flagellins, *fliC* and *fljB*. These isogenic mutants are amotile (indicated as M^−^) and lack monomeric flagellins as direct stimuli of inflammasome activation (indicated as **F^−^**; Table 1). The mutation was introduced into the wild type background (WT**^M−F−^**; SL1344, *fliGHI::Tn10*) and in a mutant lacking a functional T1 apparatus (T1^**−M−F−**^; SL1344**,** Δ*invG fliGHI::Tn10*), respectively. We confirmed the lack of flagellin production by Western blot analysis (Fig. 1A).

To analyze caspase-1 activation, RAW264.7 macrophages were pretreated with LPS to up-regulate pro-IL-1β and infected with wild type (SL1344; WT) or WT^**M−F−**^. Mock infected cells or cells infected with T1^−^ (T1 apparatus is not functional) or T1^**−M−F−**^ (no flagellins expressed, T1 apparatus is not functional) served as negative controls. In order to compensate for the loss of motility of the flagella-less mutants, all assays were performed once without centrifugation and once with centrifugation, as described in Materials and Methods. As a measure for caspase-1 activation, we used two well established assays: i.) the release of lactate dehydrogenase (LDH) which occurs upon caspase-1 activation [31], [32], [33], [34], and ii.) the secretion of mature IL-1 that was measured in a bioassay as described previously ([29]; Fig. 1B and 1 C, respectively). Without centrifugation, infection with wild type *S*. Typhimurium resulted in a strong macrophage response which is in line with earlier publications (Fig. 1 B, C) [8], [9], [12], [32]. In contrast, the flagellin-deficient strain WT^**M−F−**^ was not able to trigger the release of LDH or secretion of mature IL-1, respectively. However, when bacteria were spun down onto the cells at the beginning of infection to compensate for the motility defect of the WT^**M−F−**^ and T1^**−M−F−**^ mutants, WT^**M−F−**^ induced a strong release of LDH and mature IL-1 (Fig. 1B,C). LDH release levels were slightly lower (p<0.05; Fig. 1B) while IL-1 release did not differ significantly from wild type *S*. Typhimurium (p≥0.05; Fig. 1C). Similar results were obtained with a mutant harbouring direct deletions of both flagellin genes, *fliC* and *fljB* (data not shown). In contrast, neither T1^−^ nor T1^**−M−F−**^ were able to induce a macrophage response (p<0.05; Fig. 1 B, C). This defect could not be rescued by centrifugation, demonstrating that a functional T1 apparatus is required for caspase-1 activation. Importantly, caspase-1 activation by the flagellin-deficient strain WT^**M−F−**^ still takes place, indicating a significant role of a flagellin-independent activation mechanism of the inflammasome, presumably by T1 and/or SopE of *S*. Typhimurium.

Since centrifugation was able to restore caspase-1 activation by flagellin-deficient *S.* Typhimurium mutants, we infered that a motile phenotype might be required for full caspase-1 activation rather than the presence of flagellin monomers. To test this hypothesis, we also included a mutant in our experiments that lacks the hook-filament junction protein FlgK which is required for assembly of flagellin monomers into the outer filament of the flagellum (Homma, Yamaguchi, 1984). Therefore, Δ*flgK* (M−F+) mutants are amotile but nevertheless express flagellin at levels comparable to wild type bacteria, but secrete higher amounts of monomeric flagellin into the culture medium (Fig. 1 A). Interestingly, the mutant T1^**−M−F+**^ (expressing flagellin, but lacking functional flagella) that was unable to secrete SopE also retained a small amout of flagellin within the bacterial cell (Fig. 1 A). This might be due to the fact that, in addition to secretion via the flagellar apparatus, a proportion of flagellin may be secreted via T1 [10].

We tested the Δ*flgK* mutant in the wild type background, WT^**M−F+**^, for its ability to induce release of LDH and mature IL-1 from RAW264.7 macrophages (Fig. 1 B, C). When WT^**M−F+**^ was added to the cells without centrifugation, this strain was not able to induce a macrophage response. However, when the defect in motility was rescued by spinning bacteria onto the cells, WT^**M−F+**^ elicited wild type levels of LDH and mature IL-1 release (p≥0.05; Fig. 1 B, C). These results show that the motility defect shared by the Δ*flgK* (WT^**M−F+**^) and the Δ*fliGHI* (WT^**M−F−**^) mutants prevents activation of caspase-1. However, WT^**M−F+**^ elicited slightly higher levels of LDH release than WT^**M−F−**^, indicating that flagellin signaling may also contribute to some extent to caspase-1 activation.

### The efficiency of host cell attachment and effector protein injection correlates with caspase-1 activation

Assessing the role of flagellin in caspase-1 activation is challenging due to its pleiotropic functions. Lack of motility prevents movement of bacteria towards their target cells and results in a much lower number of bacteria attaching to cells [35]. Therefore, amotile bacteria are most likely less efficient in establishing contact with the host cell membrane which is a prerequisite for insertion of the translocation pore of their T1 system and injection of effector proteins, such as SopE. In order to address this question, we analyzed attachment of bacteria to the macrophage cell layer and, in parallel, T1-mediated effector protein tranlsocation. The efficiency of effector translocation was monitored by using isogenic *S*. Typhimurium strains expressing a SopE^M45^-TEM-1 fusion protein instead of wild type SopE (WT_TEM_). When SopE^M45^-TEM-1 is injected into the host cell cytoplasm, the conversion of the fluorescent substrate CCF2-AM by the TEM-1 beta-lactamase can be quantified, reflecting the amount of translocated SopE fusion protein (see material and methods).

**Figure 2:**
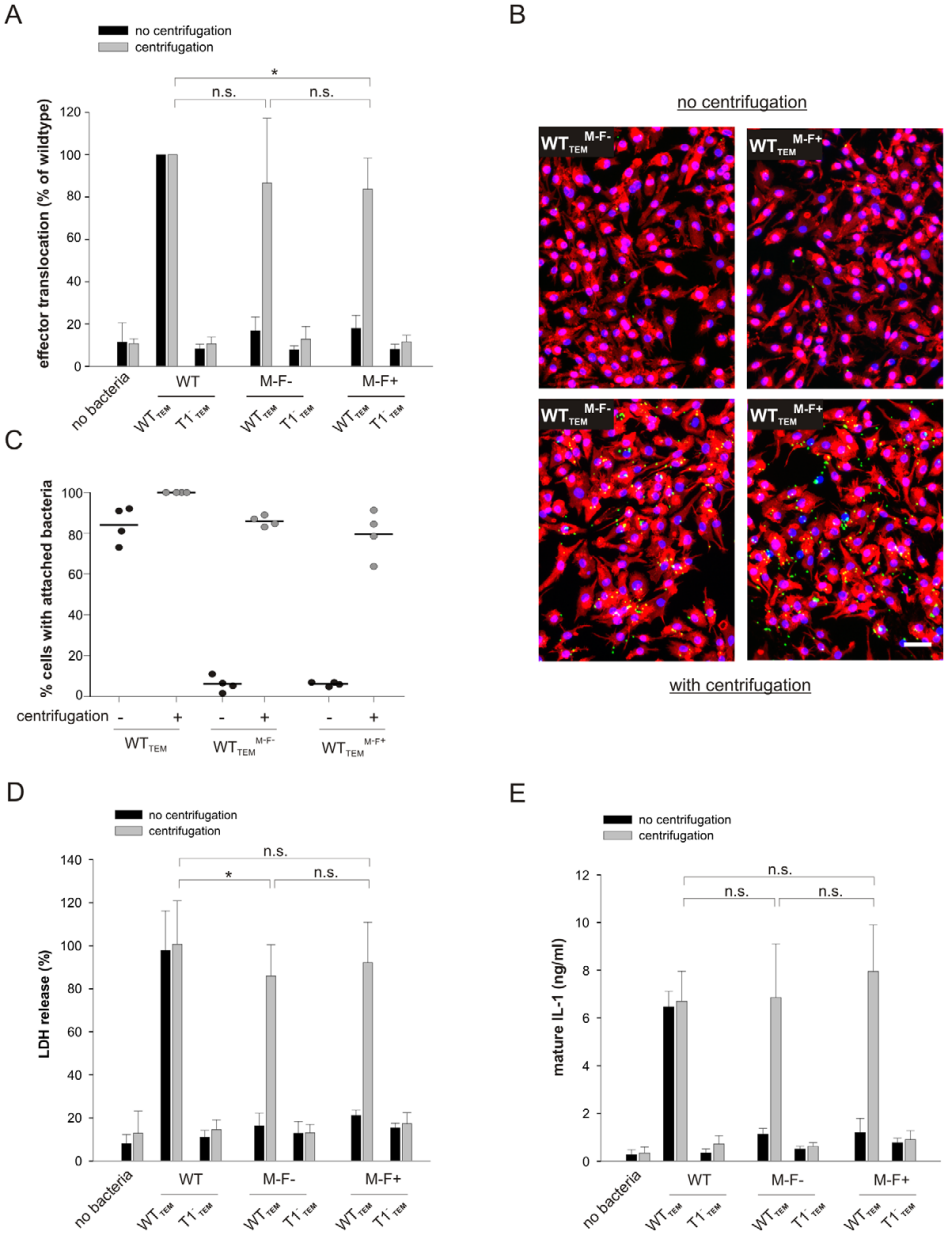
Motility defect but not lack of flagellin leads to failure in caspase-1 induction. **A**–**E**) LPS-primed RAW264.7 macrophages were infected with or without centrifugation with different strains of *S*. Typhimurium (MOI 150) that have *sopE* substituted by *sopE^m45^-tem-1*. WT_TEM_ or T1^−^_TEM_ either have normal flagella (wildtype flagella), lack flagellin expression (M−F−), or express monomeric flagellin but do not assemble flagella (M−F+). **A**) SopE^M45^-TEM-1 effector translocation into RAW264.7 macrophages was detected by measuring conversion of the TEM-1 beta-lactamase fluorescent substrate CCF2-AM. Values were normalized to the WT_TEM_ strain. Centrifugation restores effector translocation by WT_TEM_^**M−F−**^ and WT_TEM_^**M−F+**^. **B**) Infection was performed with WT_TEM_^**M−F−**^ (left side) or WT_TEM_^**M−F+**^ (right side), respectively, where after cells were washed extensively, fixed and stained with DAPI (blue), phalloidin-TRITC (red), and anti-Salmonella LPS antibody (green) to visualize attachment of bacteria. Cells with attached WT_TEM_^**M−F−**^ or WT_TEM_^**M−F+**^ without (upper panels) or with centrifugation (lower panels), or with WT_TEM_, were quantified as shown in C). Scale bar: 50 µm. **C**) Black circles: not centrifuged; grey circles: with centrifugation. Data shown from two independent experiments performed in duplicate. Black bar: mean of four data points. **D**) LDH release and **E**) IL-1 maturation after infection without (black bars) or with centrifugation (grey bars). Experiments were performed in triplicate; mean +/− SD.; n.s.: not significant; *: p-value ≤0.05.

We introduced a SopE^M45^-TEM-1 fusion into the background of the wild type, WT^**M−F−**^, and WT^**M−F+**^, respectively, yielding the strains WT_TEM_, WT_TEM_^**M−F−**^ (Δ*fliGHI*), WT_TEM_^**M−F+**^ (Δ*flgK*). As negative controls, we constructed equivalent strains with a disrupted T1 apparatus (T1^−^_TEM_, T1^−^_TEM_^**M−F−**^, and T1^−^_TEM_^**M−F+**^). LPS-pretreated RAW264.7 cells were infected with or without centrifugation (Fig. 2 A). In the absence of centrifugation, a significant change in fluorescence was observed only with the motile, T1-proficient strain WT_TEM_, indicating efficient translocation of the SopE^M45^-TEM-1 fusion protein via the T1 system (Fig. 2 A, black bars). Without centrifugation, the amotile strains WT_TEM_^**M−F−**^ and WT_TEM_^**M−F+**^ were unable to translocate SopE^M45^-TEM-1, but this defect could successfully be rescued by applying a centrifugation step (Fig. 2 A, grey bars). In contrast, T1 deficient mutants could not inject SopE^M45^-TEM-1 under all conditions.

To verify that enhanced effector translocation was due to higher attachment efficiency, we microscopically analyzed attachment of bacteria to the macrophage cell layer. LPS-pretreated RAW264.7 macrophages were infected either with or without centrifugation and were then washed extensively to remove unbound bacteria. When plates were not centrifuged, WT_TEM_^**M−F−**^ and WT_TEM_^**M−F+**^ attached only in very low numbers to the macrophages (Fig. 2 B, C). As expected, the number of bound WT_TEM_^**M−F−**^ and WT_TEM_^**M−F+**^ bacteria dramatically increased after centrifugation and was now at similar levels as the wild type (WT_TEM_) (Fig. 2 B, C).

Strikingly, higher bacterial attachment and the amount of translocated SopE^M45^-TEM-1 strongly correlated with the amounts of released LDH and secreted IL-1, respectively (Fig. 2 D, E). Equivalent to WT^**M−F−**^ and WT^**M−F+**^ that express native SopE (Fig. 1A; Fig. 1 B, C), both SopE^M45^-TEM-1 expressing strains WT_TEM_^**M−F−**^ and WT_TEM_^**M−F+**^ efficiently triggered LDH release and IL-1 maturation when a centrifugation step was applied (Fig. 2 D, E). Although T1^−^_TEM_ mutants are able to attach to macrophages (not shown), they cannot translocate SopE^M45^-TEM-1 and do not induce a caspase-1 response (Fig. 2 A, D and E). Therefore, attachment of bacteria alone is not sufficient for caspase-1 activation, but additionally requires a secretion-competent T1 system. Thus, host cell attachment, a functional T1 system, and injection of effector proteins, as measured here by translocation of SopE^M45^-TEM-1, are prerequisites for efficient caspase-1 activation in the absence of flagellin.

### SopE-stimuli and/or T1 can activate caspase-1 in the absence of flagellin in a dose-dependent manner

Our results indicated that the strength of caspase-1 activation in RAW264.7 macrophages depended on the activity of the T1 apparatus and the amount of injected effector protein, i.e. SopE^M45^-TEM-1. Thus, LDH release should correlate with the amount of injected SopE^M45^-TEM-1 in a dose-dependent manner. To test this hypothesis, we employed the *S*. Typhimurium strains SopE/E2_TEM_ (SL1344; Δ*sipA* Δs*opB*) and SopE/E2_TEM_^**M−F−**^ (SL1344; Δ*sipA* Δs*opB* Δ*fliGHI*) that both express SopE and its homolog SopE2, but lack the T1 effector protein genes *sopB* and *sipA*, encoding a phosphatidyl inositol phosphatase and an actin binding protein, respectively [36], [37]. SipA and SopB have no significant influence on caspase-1 activation in epithelial cells as well as in RAW264.7 macrophages [12]. In addition to SopE/E2_TEM_ and SopE/E2_TEM_^**M−F−**^, we used the negative control strain T1^−^_TEM_.

**Figure 3:**
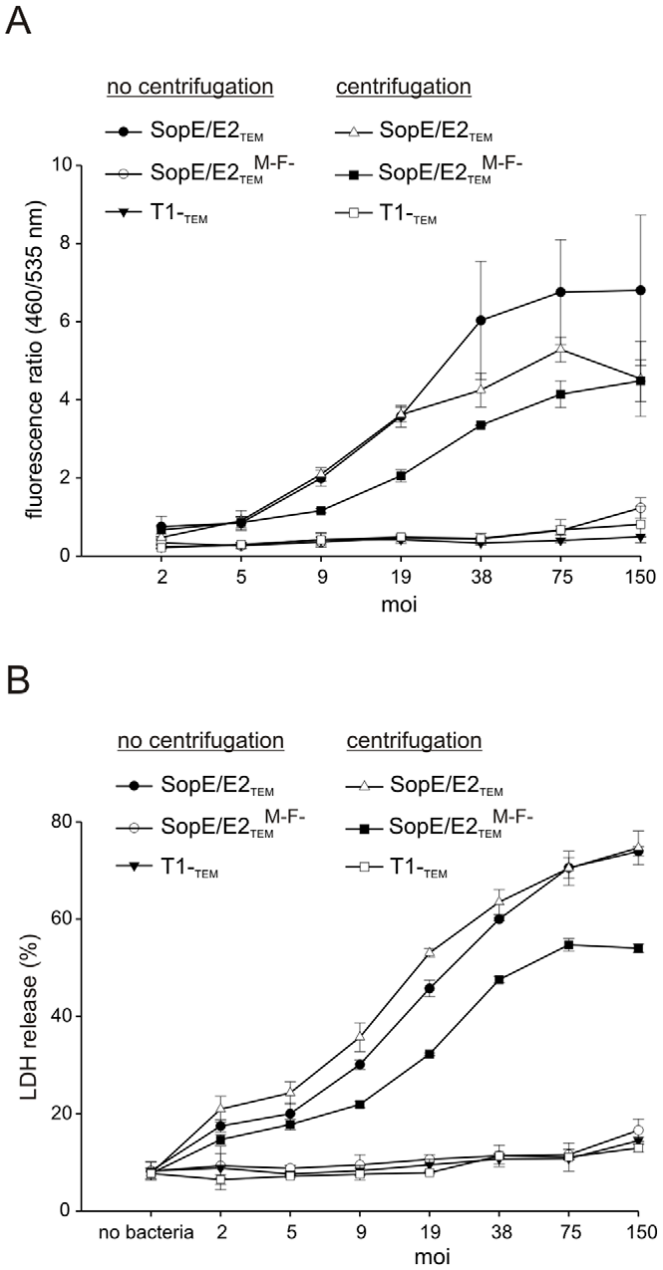
Effector- and T1-induced caspase-1 activation in the absence of flagellin is dose-dependent. **A**) SopE^M45^-TEM-1 translocation by strains SopE/E2_TEM_ (no centrifugation: black circles; centrifugation: open triangles), SopE/E2_TEM_^**M−F−**^ (no centrifugation: open circles; centrifugation: black squares), and T1^−^_TEM_ (no centrifugation: black triangles; centrifugation: open squares) at different MOI. **B**) LDH release induced by the same strains as in A) correlates with SopE^M45^-TEM-1 translocation in a dose-dependent manner. Data are representative of 3 independent experiments.

LPS-pretreated RAW264.7 macrophages were infected with a wide range of different multiplicities of infection (MOI) and we measured both the amount of injected SopE^M45^-TEM-1 fusion protein and the release of LDH without and with centrifugation (Fig. 3 A, B). With the motile strain SopE/E2_TEM_, we observed significant effector protein injection and caspase-1 activation with and without centrifugation even at very low MOI. In the case of the amotile mutant SopE/E2_TEM_^M−F−^, centrifugation was required in order to achieve effector protein injection and caspase-1 activation. If centrifuged, this amotile strain SopE/E2_TEM_^M−F−^ achieved almost the same level of effector protein injection and caspase-1 activation as the isogenic motile strain SopE/E2_TEM_. Remarkably, with both strains, the release of LDH correlated over the whole range of MOIs with the amount of injected SopE^M45^-TEM-1. The observed dose-dependence of the caspase-1 response indicates that the number of inserted T1 translocons, the amount of translocated SopE^M45^-TEM-1, or both determine the strength of caspase-1 activation in the absence of flagellin.

### SopE catalytic activity and the T1 apparatus contribute to caspase-1 activation in RAW264.7 macrophages

We could show before that SopE is a key effector inducing a caspase-1 response in RAW264.7 macrophages by flagellin-expressing *S*. Typhimurium [12]. However, it remained unclear whether SopE and/or SopE2 were able to activate caspase-1 in the absence of flagellin signaling. If over-expressed, SopE and its homolog SopE2 are each sufficient for activating caspase-1. However, if injected via T1, SopE is a potent activator of caspase-1, whereas a strain expressing SopE2 but lacking SopE, SipA, and SopB (Δ*sipA* Δs*opB* Δ*sopE*) does not significantly contribute to caspase-1 activation [12]. Both effector proteins are GEFs for RhoGTPases and show 70% identity [13], [38], [39].

**Figure 4:**
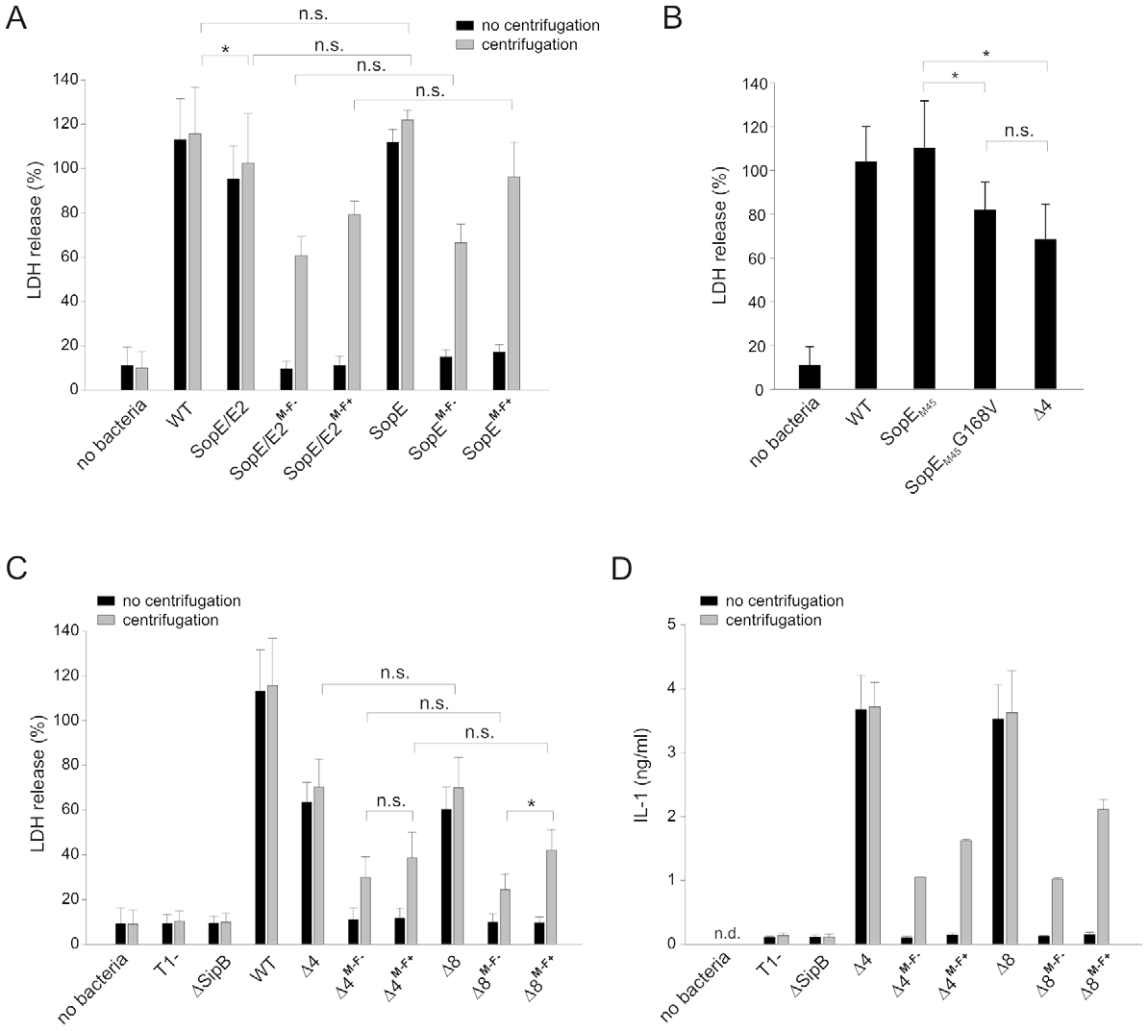
SopE and an intact T1 system contribute to flagellin-independent caspase-1 activation. **A**) SopE is the main effector protein mediating caspase-1 activation in the absence of flagellin. LDH release induced by strains expressing SopE and SopE2 (Δ*sipA* Δ*sopB;* SopE/E2, SopE/E2^**M−F−**^, and SopE/E2^**M−F+**^) is equivalent to LDH release induced by strains additionally lacking SopE2 (Δ*sipA* Δ*sopB* Δ*sopE2;* SopE/E2, SopE/E2^**M−F−**^, and SopE/E2^**M−F+**^). Note that data shown in A) and C) were obtained from the same experiments. The value for WT in A) was replotted in C) for better comparison. **B**). The catalytic activity of SopE (infection with SopE^M45^ strain) is required for full LDH release. A strain with a catalytically inactive SopE mutant (SopE^M45^G168V; Δ*sipA* Δ*sopB* Δ*sopE2*) induces the same level of LDH release as a mutant lacking four effector proteins including SopE (Δ4; Δ*sipA* Δ*sopB* Δ*sopE* Δ*sopE2*). **C**) Mutants lacking four (Δ4; Δ*sipA* Δ*sopB* Δ*sopE* Δ*sopE2*) or eight (Δ8; Δ*sipA* Δ*sopB* Δ*sopE* Δ*sopE2* Δ*sopA* Δ*sptP* Δ*spvB* Δ*spvC*) virulence proteins induce LDH release with (Δ4^**M−F+**^, Δ8^**M−F+**^) or without flagellin (Δ4^**M−F−**^, Δ8^**M−F−**^), whereas a *sipB* mutant that lacks the ability for translocon insertion does not. **D**) IL-1 maturation induced by Δ4, Δ8, Δ4^**M−F+**^, Δ8^**M−F+**^, Δ4^**M−F−**^, and Δ8^**M−F−**^. n.d.: not detected. Mean +/− standard deviation of triplicates from at least 2 independent experiments. n.s.: not significant; *: p-value ≤0.05 (paired t-test in panel B; Mann-Whitney U test in panel C). Data shown in D) are representative of 3 independent experiments.

Here, we compared the ability of SopE and SopE2 to elicit a caspase-1 response in the presence or absence of flagellin, respectively. To that end, we constructed a strain expressing native SopE and its functional homolog SopE2, but lacking the T1-secreted effectors SipA and SopB (strain SopE/E2; Δ*sipA* Δs*opB*), and a strain with additional deletion of *sopE2* (strain SopE; Δ*sipA* Δs*opB* Δ*sopE2*). In the background of the strains SopE/E2 and SopE, we additionally deleted *fliGHI* or *flgK*, yielding the strains SopE/E2^**M−F−**^ and SopE/E2^**M−F+**^, and SopE^**M−F−**^ and SopE^**M−F+**^, respectively. The strain SopE/E2 leads to release of LDH and IL-1 from RAW264.7 macrophages to an extent similar to the wildtype (Fig. 4 A). Without centrifugation, the amotile strains SopE/E2^**M−F−**^ and SopE/E2^**M−F+**^ did not induce a caspase-1 dependent response, whereas centrifugation partially restored caspase-1 activation by these strains (Fig. 4 A). Similar results were obtained with the strains that lack SipA, SopB, and additionally SopE2, expressing exclusively SopE out of the four major T1 effector proteins (strains SopE, SopE^**M−F−**^, SopE^**M−F+**^, Fig. 4 A), showing that SopE2 does not further enhance caspase-1 activity. With centrifugation, activation by SopE took place in the absence of flagellin (Fig. 4 A, grey bars). These results verified that SopE, and not SopE2 contributes to caspase-1 activation in RAW264.7 cells, and that caspase-1 activation by SopE is independent of flagellin.

We have shown before that transfection of HEK293T cells with wild type SopE, but not with a catalytically inactive mutant can trigger cleavage and activation of caspase-1 [12]. In order to test if the catalytic activity of SopE also plays role in *S*. Typhimurium-induced caspase-1 activation in RAW264.7 macrophages, we compared the strain SopE^M45^ (Δ*sipA* Δ*sopB* ΔS*opE2 sopE::sopE^M45^*) with the catalytically inactive mutant SopE^M45^G168V (Δ*sipA* Δ*sopB* ΔS*opE2 sopE::sopE^M45^G168V*). SopE^M45^ strongly induced the release of LDH, while LDH release was significantly reduced after infection with the inactive mutant SopE^M45^G168V (p<0.05; Fig. 4 B). Remarkably, LDH release by the SopE^M45^G168V inactive mutant did not differ significantly from the mutant lacking four effector proteins, including SopE (p≥0.05; strain Δ4; Δ*sopE* Δ*sipA* Δ*sopE2* Δ*sopB*; Fig. 4 B), arguing that the catalytic activity of SopE is required for full-blown caspase-1 activation. Please note that SopE^M45^G168V and Δ4 both harbor a functional T1 system, other T1 effectors and flagella and that these account for the remaining LDH release activity in RAW264.7 macrophages.

For analyzing additional putative caspase-1 activation factors present in the Δ4 mutant independently of flagellin, we introduced additional deletions of *fliGHI* or *flgK* into the Δ4 strain, yielding Δ4^**M−F−**^ and Δ4^**M−F+**^, respectively. Moreover, we constructed Δ8 mutants lacking two further effector proteins as well as SpvB and SpvC (Δ*sopE* Δ*sipA* Δ*sopE2* Δ*sopB* Δ*sopA* Δ*sptP* Δ*spvB* Δ*spvC*), and additionally *fliGHI* (Δ8^**M−F−**^) or *flgK* (Δ8^**M−F+**^), respectively. Again, LPS-pretreated RAW264.7 cells were infected either without or with centrifugation. All corresponding Δ4 and Δ8 strains yielded equivalent results (p≥0.05; Fig. 4C), indicating that *sopA*, *sptP*, *spvB*, and *spvC* did not significantly contribute to caspase-1 activation in RAW264.7 cells. Flagellin expression tended to enhance caspase-1 activation since LDH release induced by the Δ8^**M−F+**^ mutant was stronger than by the mutants lacking flagellin expression (p<0.05 vs. Δ8^**M−F−**^; Fig. 4 C). These observations were corroborated by the IL-1 release assay (Fig. 4D). However, even in the absence of flagellin, both the Δ4^**M−F−**^ and the Δ8^**M−F−**^ mutants were still able to induce a significant caspase-1 dependent response (p<0.05 vs. T1^−^), although this response was much weaker than with the SopE-expressing, flagellin-deficient strain SopE^**M−F−**^ (Fig. 4 A). Interestingly, a mutant lacking SipB (which is necessary for formation of the T1 translocon pore) or a T1^−^ mutant lacking the whole T1 secretion apparatus did not induce any LDH release (Fig. 4 C). Although we cannot exclude that other effector proteins or bacterial factors might play an additional role, our data suggest that in absence of flagellin and SopE, the translocon of the T1 system itself is sensed by the macrophage, resulting in activation of caspase-1.

## Discussion

*S*. Typhimurium is thought to activate caspase-1 in macrophages by different types of stimuli, including flagellin, the T1 translocon and the T1 effector protein SopE [8], [9], [10], [11], [12]. Here, we have compared the contributions of these stimuli to caspase-1 activation in RAW264.7 macrophages. SopE and the T1 translocon accounted for most of this stimulation. Flagellin was a weak caspase-1 activating stimulus in RAW264.7 cells. Much rather, it was required for efficient movement and binding to the host cell.

Inflammasome activation by bacterial flagellin has been demonstrated for several bacterial species, such as *S*. Typhimurium, *Pseudomonas aeruginosa*, and *Legionella pneumophila* [8], [9], [10], [40], [41]. These findings were challenged by more recent data reporting inflammasome activation by flagellin-deficient bacteria [11], [42], [43]. Until now, it has not become clear whether the contradictory results actually reflect two distinct mechanisms of inflammasome activation: one depending on monomeric flagellin, and another one acting independently of flagellin.

In the present work, we investigated the requirements for flagellin-dependent as well as -independent activation of caspase-1 by *S*. Typhimurium in RAW264.7 macrophages. We found that a *S*. Typhimurium mutant deficient in flagellin expression (Δ*fliGHI*, M−F−) failed to activate caspase-1 due to its motility defect resulting in inefficient contact with the macrophages. This defect was almost completely compensated when host cell contact was enhanced by applying a mild centrifugal force facilitating attachment of bacteria to the macrophages. These results show that motility mediated by the flagella, but not necessarily presence of flagellin molecules (as proposed by earlier reports) is required to efficiently trigger inflammasome activation. This conclusion is further supported by the results obtained with the flagellin-expressing but non-motile Δ*flgK* mutant (M−F+). This mutant also failed to attach efficiently to host cells and to activate caspase-1. Centrifugation could restore caspase-1 activation by Δ*flgK* mutants, although in some cases not to the level of corresponding motile strain. This might be due to the fact that centrifugation is a means to enhance contact between bacteria and host cells, but does not fully substitute for functional flagella which might also increase attachment efficiency.

Although caspase-1 activation can occur in the absence of flagellin, our results suggest that flagellin can act as a weak inflammasome activating factor in RAW264.7 cells, as was shown before [8], [9], [10]. In fact, some Δ*flgK* mutants (e.g. SopE^**M−F+**^) often induced a slightly stronger caspase-1 dependent response than the corresponding Δ*fliGHI* mutants (e.g. SopE ^**M−F+**^), supporting an activating role of flagellin. However, the ability to move to and intimately attach to the target cell seems to play a more important role in caspase-1 activation by *S*.Typhimurium.

Besides flagellin function, we analyzed other factors contributing to caspase-1 activation in RAW264.7 macrophages. We have shown before that the T1-secreted effector protein SopE plays a role in caspase-1 activation [12]. In RAW264.7 macrophages, strains producing SopE trigger a caspase-1 mediated response almost as efficiently as the wild type (Fig. 3 A, B). Mutants of these strains which additionally lack *fliGHI* (M−F−) and therefore do not express flagellin are still capable of activating caspase-1 when their motility defect is compensated by centrifugation. Thus, SopE potently activates caspase-1 independently of flagellin. Flagellin-independent caspase-1 activation by *S*. Typhimurium was reported to require increased multiplicities of infection. For example, Miao et al. report that a Δ*fliC*Δ*fljB* mutant induces IL-1β release by bone marrow macrophages only after increasing the MOI from 5 to 80 [8]. In RAW264.7 macrophages, we found that LDH release was induced by SopE/E2_TEM_^**M−F−**^ even at low multiplicities of infection (∼10). This higher sensitivity of RAW264.7 cells towards flagellin-independent capsase-1 activation was unexpected as BMDM are thought to be more sensitive to *S*. Typhimurium infection than RAW264.7 macrophages. Currently, we cannot explain this discrepancy, although differences in the experimental setup (centrifugal force applied, time of infection) or use of *S.* Typhimurium strain SL1344 in our study versus strain ATCC14028s (naturally lacks *sopE*) in the other might partially account for it. Nevertheless, *S*. Typhimurium can clearly activate caspase-1 in the absence of flagellin, and SopE contributes significantly to this effect.

Earlier reports suggest that the T3SS of pathogenic bacteria itself may lead to inflammasome activation [11], [41], [45], [46]. Effector translocation cannot be completely distinguished from the action of a functional, secretion competent T1 system (inserted translocon), because increasing the MOI increases both the number of inserted translocons and the amount of translocated effector. We addressed this issue by deleting up to 8 effector proteins. This did not reduce LDH release below the levels obtained with the catalytically inactive SopE variant (SopE^M45^G168V), whereas a functional T1 system was required for caspase-1 activation, as indicated by the lack of caspase-1 activation by *invG* or *sipB* mutants. This supports the notion that the secretion-competent T1 system of *S*. Typhimurium triggers caspase-1 activation. Importantly, activation by the multiple effector knockout mutants even occurs when flagellin is not present, as demonstrated by the strains Δ4^**M−F−**^ and Δ8 ^**M−F−**^. Therefore, the stimulus provided by the T1 system itself is clearly independent of SopE and flagellin. However, we cannot completely exclude that one of the remaining effector proteins or another bacterial factor that is translocated via the T1 system exerts an additional stimulation.

The mechanism explaining caspase-1 activation by the T3SS translocon has attracted significant interest. Recently, PrgJ which is a component of the inner rod of the basal body of T1, was identified as a factor activating caspase-1 via the NLRC4/IPAF inflammasome [11]. PrgJ that shares homology with FliC and *prgJ* transfection into macrophages resulted in caspase-1 activation and IL-1β secretion, whereas a *S*. Typhimurium *prgJ* mutant did not activate caspase-1. However, there was no evidence shown for direct translocation of PrgJ from the bacteria into the host cell cytoplasm. Like the Δ*invG* mutant (T1^−^) used in our study, a *prgJ* mutant cannot assemble a secretion competent T1 apparatus and lacks the capacity to insert a translocon into the host cell membrane. Therefore, it is difficult to conclude whether leakage of PrgJ through the T1 needle (as was proposed for FliC; [10]) or rather the physical interaction of the needle tip with the host cell, or both trigger inflammasome activation. For *Yersinia enterocolitica*, pore formation by the T3SS system was observed and resulted in caspase-1 activation in macrophages and HeLa cells, and the tip protein YopB was required for this effect [45]. YopB is a close homolog of SipB, a protein which is necessary for translocon formation by the T1 system of *S*. Typhimurium. Although SipB was shown to interact with and activate caspase-1 [47], we have not been able to induce caspase-1 activation by transfection of SipB [12]. Further work is needed to understand how T3SS translocon insertion results in caspase-1 activation.

Clearly, numerous pathogens can employ T3SS for inflammasome activation and the induction of caspase-1 dependent pro-inflammatory responses. Interestingly, there seems to be a significant diversity in the types of stimuli affecting caspase-1 by these different pathogens. The expression of the T3SS is the only common denominator. This suggests that the T3SS translocon itself represents a conserved inflammasome activating stimulus and that pathogen-specific stimuli have evolved to further modulate this effect. If this were the case, we may expect to find numerous additional inflammasome activators in different strains of T3SS pathogens and in new species bearing this virulence system. This will be an interesting topic for future research and a step towards a “unifying” model for caspase-1 activation by T3SS bearing pathogens.
